# Moving the field: the sensorimotor perspective on autism (Commentary on “Rethinking autism: implications of sensory and motor differences,” an article by Anne Donnellan, David Hill, and Martha Leary)

**DOI:** 10.3389/fnint.2013.00006

**Published:** 2013-02-21

**Authors:** Ralph J. Savarese

**Affiliations:** Humanities Writ Large Fellow, Duke Institute for Brain Sciences, Duke UniversityDurham, NC, USA

This triad of clinicians and researchers has been advancing a sensorimotor perspective on autism for years; at last, the scientific community is beginning to catch up. Since the article's initial publication in *Disability Studies Quarterly*, the sensorimotor hypothesis has garnered even more support (Donnellan et al., 2010). For example, a meta-analysis from 2010 concluded, “ASD is associated with significant and widespread alterations in motor performance” (Fournier et al., 2010). The article went so far as to propose that motor differences constitute a “core element” of autism and that “interventions aimed at improving … motor coordination (i.e., gait and balance, arm functions, and movement planning)” should be considered. A study from 2011 found that gross and fine motor differences in autistic children increased significantly with “each 6-month period of chronological age” (Lloyd et al., 2011). It recommended “addressing motor development in early intervention treatments.” And a study from 2012 reported that “motor skills were substantially impaired among ASD-affected children and highly correlated with autistic severity and IQ” (Hilton et al., 2012). By looking at the siblings of autistic children and finding in them no equivalent impairment, the study was able to directly link sensorimotor disturbances with ASD. It, too, contended that motor impairment is a “core characteristic” of autism and that treatment should reflect this fact. The tide has clearly shifted with respect to the sensorimotor hypothesis; what was once dismissed out of hand by an earlier generation of autism researchers is now increasingly being taken up for its superior explanatory power.

One of the many virtues of “Rethinking autism: implications of sensory movement differences” is the elaborate qualitative context in which the authors situate the scientific research they cite. Appealing to the rich autobiographical literature that has emerged over the last 20 years, they remind us of the danger in interpreting what professionals disparagingly refer to as autistic “behaviors.” “Differences in the way people are able to use their bodies and focus their attention,” they write, “lead many to assume that a person does not care to participate or communicate and does not desire relationship.” This assumption has been especially devastating for so-called “low-functioning” autistics whose sensorimotor challenges, we can now say with confidence, are acute. It has saddled them with all manner of stigmatizing judgments—from impaired imagination to impaired empathy to impaired reasoning abilities. Accounts by self-advocates have repeatedly stressed a difficulty, on the one hand, suppressing non-volitional movements, and, on the other, instigating and sustaining purposeful ones. More basically, they have exhibited sophisticated, and at times intensely lyrical, introspection, which, according to the *DSM*, should not be possible. Yet despite what self-advocates have been saying in books, articles, films, and on the Web, experts continue to interpret atypical comportment as the outward sign of inward dysfunction.

Research sensitive to the sensorimotor hypothesis has revealed a very different picture, however. For instance, a study from 2005 argued that empathy is “not a unitary system” but rather three “partially dissociable systems”: emotional, cognitive, and motor (Blair, 2005). Autistics, it turns out, have no trouble at all with the first but struggle, on average, with the second two. Describing autism as a difficulty attaching words to emotional states and motorically executing an expected response is very different from describing it as a lack of feeling for other people. The autobiographical literature is replete with accounts of autistics “fusing” with the pain of others, so completely do they experience it, or of needing time to organize their thoughts and bodies in the face of such an emotional onslaught (Savarese, 2010a). That neuroscientists tend to denigrate emotional empathy as “lower-order” processing should not discourage us from identifying it as an autistic *strength*; indeed, it could well be that cognitive empathy requires the diminishment of feeling and the distancing of the empathetic subject from the person in pain.

Consider how one prominent autist describes listening to a report about a coalmining disaster on TV:

I see these stories, sometimes in vermillion or indigo, the richness depending upon the intensity of the stories. Sometimes they smell like vitriol and sometimes they smell like boiling starch in a pot of clay. And sometimes they have the essence of the twilight sky.

As I feel my worries for the trapped coal miners, I can smell the boiling starch, frothing on the brim of the clay pot, then spilling out with the smell of burning rice. My worries grow as the voice of the newsreader continues to say that the miners are still trapped. I smell burning rice spread across the room as more starch spills out ….

My body begins to itch as though tiny black tickle ants have been set free from a box. They can smell the burning rice from the spilling starch, and they rush around to find the source with a collective ant hunger. My worry now accumulates in and across my itching skin, as the voice of the newsreader comes from far away, like a blue floating balloon. I have no hold on it because it floats away, leaving me with itchy skin (Mukhopadhyay, 2008).

The author of three well-received books and the subject of a *60 Minutes* profile, Tito Mukhopadhyay has been labeled “severely” autistic by the medical community. He cannot speak, he experiences significant anxiety, he stims, and yet he is extraordinarily well read, and he has learned, after much practice, to express himself by writing, or typing independently. He has never been allowed in a regular school—in fact, he once responded to an interviewer's question about his education by typing, “My school is the doubt in your eyes.” For the last 5 years or so, I have been mentoring Tito, Skyping him into my literature and creative writing classes at Grinnell College, commenting on his poems and stories. This year, while I am on fellowship at Duke University's Institute for Brain Sciences, we are reading *Moby Dick* together by Skype.

In the above passage, Tito makes clear just how much feeling he has for the predicament of the miners and just how debilitating such feeling is. Alternative sensory processing completely overruns his ability to manage what he hears: the effect of the words paradoxically threatens the words themselves—at least during the period of their registration. Later, of course, Tito is able to chronicle his embodied response and to do so in prose rivaling that of professional writers. When empathy is this overwhelming, purposeful empathetic response becomes impossible. Notice the gap between what is actually going on inside of Tito and what an observer would likely conclude about his behavior: that he is acting strangely, that he is oblivious to the suffering of others. It is also worth remembering the insights of the neurodiversity movement: empathy comes in many forms. I have always found it ironic that in his famous profile of Temple Grandin, Oliver Sacks failed to acknowledge his own alienation from the animal world, though he was interviewing an internationally accomplished cattle expert and though he was dissecting—one might even say, perseverating about—Grandin's partial alienation from the human one (Sacks, 1995). Thinking differently about difference makes room for a plethora of empathetic strengths, not the rigid and self-congratulatory normalization of one.

Another virtue of “Rethinking autism: implications of sensory movement differences” is its broader consideration of movement disorders. Reflecting on ASD in the light of *encephalitis lethargica* or Parkinson's can help us to understand otherwise cryptic accommodations to an alternative neurology; it can also help us to develop more effective therapies. The behaviors that experts tend to read psychologically may instead be a general adaptive mechanism. The human organism depends on sensory input to interpret the external physical world in a consistent and reliable manner, and on somatosensory-motor input to act on that interpretation, also in a consistent and reliable manner. When those sources of external and internal inputs are absent or disturbed, no stable percept can emerge. The organism searches and searches for what it needs and tries to preserve the minimal consistency it has found (hence, the familiar insistence on sameness in autism). This all-consuming process affects both the cortical and subcortical areas of the brain, as the research that the authors cite demonstrates. And it quickly takes on a bio-cultural cast, alienating the autist from the enriching social interaction that every one of us needs to develop. As a young child, Tito used to spin furiously because his body felt so scattered; this adaptive habit, like his retreat from synesthetic overstimulation in response to strong emotion, left him vulnerable to misinterpretation and made it exceedingly difficult to convince people that he belonged in a regular school.

Because each autist will compensate for his differential development in a unique way, no two individuals with the same observational score of ASD will have the same manifestations of the disorder. This fact highlights the importance of personalized diagnosis, treatments, and tracking of progress—a clear choice outlined in the paper. And yet, the root disturbance of ASD—sensorimotor dysfunction—should frame such an individualized approach. The article concludes by referencing Jamie Burke, a senior at Syracuse University, who at the age of 13 began to learn how to speak while typing (independently) on his augmentative communication device. An innovative occupational therapist used a range of movement therapies to coax a voice from Jamie's fingertips. At first, he could only speak while typing; then he could only read aloud something that he had typed, the memory of having produced the words with his fingers somehow guiding his mouth. Now he can read aloud another person's text and even speak without first typing what he wants to say. When he is nervous, however, he still prefers to prime his voice motorically, as he did when the two of us were interviewed on Iowa Public Radio as part of a show about the neurodiversity movement (Kieffer, 2012). It was the first live radio interview with a formerly non-speaking autist—at the beginning, the show's host explained to the audience that it would be hearing the sound of a keyboard before Jamie spoke. And then together we all talked about a different way of looking at autism.

To facilitate more fluid typing, Jamie regularly used a metronome, a therapy that Parkinson's patients use to overcome their own movement challenges. In a published interview with me, Tito all but says that William Blake, the eighteenth century British poet, taught him how to tie his shoes (Savarese, 2010b). Wrapping the tetrameter of a beloved poem around his fingers, he coaxed them to execute the necessary movements. We know that listening to a metrical poem activates the listener's motor systems (Aleman and van't Wout, 2004). A recent study revealed that listening to unfamiliar music activates them, too (Rauschecker et al., 2012). Even more intriguing, the interstices between songs on a familiar CD do the same. The researchers hypothesized that motor areas support sequential mastery and, in the process, provide a memory boost. This is why we all know in advance which song is coming next on our favorite albums! It is as if our motor systems create an essential continuum by constantly anticipating—we might even say, by constantly remembering—the future. Perhaps for Jamie and Tito, respectively, the metronome and the tetrameter served as a kind of rhythmic prosthesis or taxi, compensating for inadequate motor guidance and bridging the CD-like gaps in complex tasks such as typing or tying one's shoes. By considering the implications of sensorimotor differences in autism, we can begin to rescue autistics from the sub-human status we have assigned them and, with their help, craft a more inclusive and empowering society.
